# Xanthogranulomatous Osteomyelitis Presenting as Swelling in Right Tibia

**DOI:** 10.1155/2011/257458

**Published:** 2011-11-17

**Authors:** Girish Kamat, Vandana Gramapurohit, Aneel Myageri, Chidendra Shettar

**Affiliations:** ^1^Department of Pathology, SDM College of Medical Sciences and Hospital, Manjushree Nagar, Dharwad 580009, India; ^2^Department of Orthopedics, SDM College of Medical Sciences and Hospital, Dharwad 580009, India

## Abstract

Xanthogranulomatous osteomyelitis is a very rare form of chronic osteomyelitis which presents like a bone tumor. Three cases of xanthogranulomatous osteomyelitis have been described previously in the literature. We present a case of xanthogranulomatous osteomyelitis occurring in the distal part of tibia. This case presented as swelling in a 13-year-old boy. Simple curettage proved to be curative in the present case.

## 1. Introduction

Xanthogranulomatous reaction is a rare form of chronic inflammation characterized histologically by presence of high number of foamy histiocytes admixed with lymphocytes and plasma cells [1]. Cases of xanthogranulomatous inflammation have been described in various organs such as kidney, gallbladder, colon, pancreas, and salivary gland [2]. Only 3 cases of xanthogranulomatous inflammation involving bone have been reported so far in the literature [1, 3]. We report a case of xanthogranulomatous osteomyelitis which presented as a swelling in the lower end of the right tibia. This case is being reported on account of its extreme rarity.

## 2. Case Report

**Figure 1:**
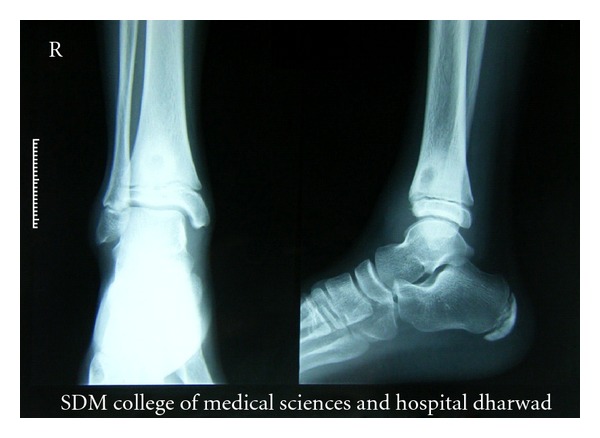
X-ray of right ankle joint showing submetaphyseal osteolytic lesion with sclerotic margin, seen in distal end of right tibia.

A 13-year-old boy presented to Department of Orthopedics with pain and swelling in the right ankle. Swelling had appeared about 1 year back and was gradually increasing in size. Examination revealed bony thickening over the lateral malleolus. Ankle and subtalar joints had full range of mobility. Parameters of complete blood count were within normal range. ESR was 68 mm at 1st hour. Plain radiography revealed a submetaphyseal lytic lesion in the distal tibia with a sclerotic margin around it (Figure 1). With these findings, a diagnosis of Brodie's abscess was made. Curettage was done under spinal anesthesia through the anterior approach. Curetted material was sent for histopathological and microbiological assessment. 

**Figure 2:**
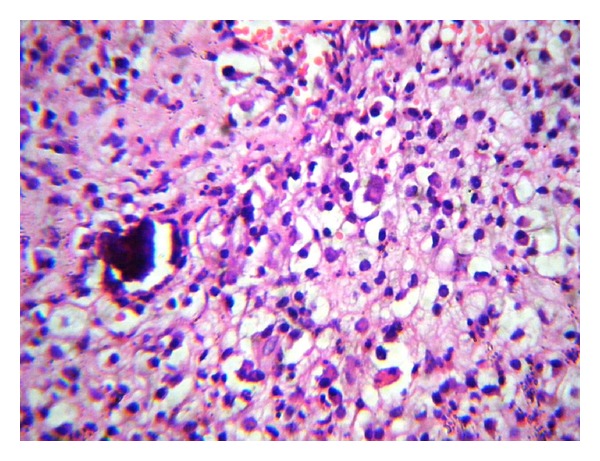
Sheets of foamy histiocytes admixed with lymphocytes and plasma cells. A necrotic bone fragment is also seen (H and E, 400x).

 On gross examination, specimen consisted of multiple, soft, gray brown to gray white tissue bits, with largest measuring 0.5 × 0.5 × 0.3 cm. Histopathology showed small irregular necrotic bony fragments and degenerated cartilage, surrounded by mixed inflammatory cell infiltration consisting of plenty of foamy histiocytes along with lymphocytes and plasma cells (Figure 2). Few neutrophils and occasional histiocytic giant cells were also seen. Fibrosis was present. All these features were consistent with xanthogranulomatous osteomyelitis. From the tissue sent for culture, staphylococcus aureus was isolated. PCR study for Mycobacterium tuberculosis was negative. Patient recovered completely after the surgery.

## 3. Discussion

Xanthogranulomatous osteomyelitis is a close mimicker of neoplastic lesions of bone. In fact, xanthogranulomatous inflammation in various organs forms a mass lesion giving an appearance of tumor [1]. Histology of this lesion shows infiltration of chronic inflammatory cells, along with plenty of foamy histiocytes. These histiocytes are large cells containing abundant, granular, eosinophilic cytoplasm.

Patients of previously described 3 cases were aged 5 years, 14 years, and 50 years. Two cases were seen in boys and one was seen in a postmenopausal lady. Sites involved in these cases were first rib, tibial epiphysis, and ulna [1, 3].

Microscopic differential diagnoses for xanthogranulomatous osteomyelitis are Langerhan's cell histiocytosis, Erdheim-Chester disease, and lipid storage disorders. Langerhan's cell histiocytosis commonly affects mid-shaft portions of long bones and often has infiltration of eosinophils. Erdheim-Chester disease is a multifocal disorder with frequent involvement of extraskeletal tissues. Histology shows presence of cholesterol clefts along with foamy histiocytes. In case of lipid storage diseases, such as Gaucher's disease and Niemann Pick disease, foam cell transformation is usually seen within bone marrow. Moreover, a clinical background often helps in differentiating storage disorders from xanthogranulomatous osteomyelitis [1].

## 4. Conclusion

This case is presented because of its rarity. There are only 3 cases of xanthogranulomatous osteomyelitis reported so far in the literature. It is very important to differentiate this lesion from bone neoplasms. A microscopic examination is essential in establishing correct diagnosis.
